# Activation of the Cholinergic Anti-inflammatory Pathway Attenuated Angiotension II-Dependent Hypertension and Renal Injury

**DOI:** 10.3389/fphar.2021.593682

**Published:** 2021-03-17

**Authors:** Shu-jie Wu, Zhe-wei Shi, Xue Wang, Fang-fang Ren, Zuo-yi Xie, Li Lei, Peng Chen

**Affiliations:** ^1^ Department of Cardiology, The Second Affiliated Hospital and Yuying Children’s Hospital of Wenzhou Medical University, Wenzhou, China; ^2^ Department of Cardiology, Zhuji Affiliated Hospital of Shaoxing University, Zhuji, China

**Keywords:** cholinergic anti-inflammatory pathway, autonomic control, AngII-induced hypertension, renal inflammation and fibrosis, GTS-21 dihydrochloride

## Abstract

**Background**: Angiotensin II (AngII) induces renal fibrosis, characterized by fibroblast proliferation, inflammatory cell infiltration and excessive extracellular matrix deposition, all of which was relevant closely to hypertension. The vagus nerve-related cholinergic anti-inflammatory pathway (CAP) modulates local and systemic inflammatory responses. The aim of present study was to determine the effect of CAP on renal inflammation and fibrosis.

**Methods and Results**: AngII-induced hypertension was induced *in vivo* by 14-days low-dose AngII infusion from osmotic minipumps. We used GTS-21 dihydrochloride, a selective nicotinic acetylcholine receptor agonist. Daily intraperitoneal GTS-21 injection and/or vagotomy started after hypertension was confirmed and continued for 4 weeks. The elevated blood pressure caused by AngII was significantly attenuated by GTS-21. Improved baroreflex sensitivity was observed after GTS-21 administration. Masson stain and immunoblotting revealed that deposition of excessive fibrosis and overexpression of inflammatory cytokines induced by AngII was reduced by GTS-21. To determine the role of autonomic control in CAP, unilateral vagotomy was performed. Vagotomy weakened the effect of CAP on AngII-induced hypertension. *In vitro*, GTS-21 suppressed NF-κB activation, attenuated AngII-induced epithelial-mesenchymal transition and reduced inflammation and fibrosis in NRK-52E cells; α-bungarotoxin (α-Bgt, an α7-nAChR selective antagonist) partly inhibited these effects.

**Conclusion:** CAP protected against AngII-induced hypertension via improvement in autonomic control, suppression of NF-κB activation, and reduction of renal fibrosis and inflammatory response.

## Introduction

Hypertension, is a major risk factor for heart attack, stroke, and kidney diseases; it is the most common chronic disease worldwide (Macmahon et al., 1990; Kearney et al., 2005). Angiotensin II (AngII) plays a critical role in contributing to the development of hypertension as well as the pathology of several cardiovascular and kidney diseases (Chappell, 2007). Massive increase of AngII modulates renal fibrosis by direct effects on the matrix and by upregulating the pro-inflammatory factors (e.g., TGF-β, TNF-α, and NF-κB) (Tang et al., 2019). Subsequently, renal fibrosis may lead to the progression of hypertension, followed by elevated glomerular capillary pressure and aggravation of renal sclerosis (Tang et al., 2019; Zhang et al., 2020). Recent data have shown that pharmacological and physiological interventions of inflammation or/and fibrosis during AngII-related renal hypertension alleviated renal injury and dysfunction of other organs (Rüster and Wolf, 2011; Simões E. Silva et al., 2013). These findings suggest that targeting inflammatory responses and fibrotic progression may attenuate kidney injury and improve the efficiency of treatment strategies, thereby inhibiting fibrosis and inflammation. This process may be a promising approach to treatment of AngII-related renal hypertension.

The pathogenesis of hypertension is complex and the precise mechanisms remain obscure. The dysfunction of autonomic control, featured by overactivity of sympathetic and reduced parasympathetic tone, is hypothesized to contribute to the development of hypertension (Dauphinot et al., 2013). The short-term regulation of blood pressure is mainly dependent on baroreflex control, while baroreflex sensitivity (BRS), recognized as a marker of autonomic function, plays an important role in the long-term development of hypertension (Huang et al., 2017). Impaired BRS and autonomic dysfunction are closely associated with essential hypertension, and the impairment of BRS predicts mortality in hypertension (Hesse et al., 2007; Carthy, 2013). Interestingly, normalizing the autonomic cardiovascular control and restoring reduced BRS in hypertensive animals reduced cardiac and renal oxidative stress, subsequently reducing peripheral blood pressure (Botelho-Ono et al., 2011; Da Silva Dias et al., 2019).

The cholinergic anti-inflammatory pathway (CAP) is an immune response regulatory mechanism that depends on the autonomic nervous system (Tracey, 2002). Signals, generated by tissue injury, infection or ischemia, reach the central nervous system through sensory afferent vagus, and then a response is returned via the efferent motoric part of the vagus nerve, referred to the inflammatory reflex (Czura and Tracey, 2005; Saeed et al., 2005). Increased parasympathetic tone has been shown to inhibit cytokine release, attenuating tissue injury and ameliorating inflammation-mediated injury in models of sepsis, colitis, and myocardial ischemic injury (Olofsson et al., 2012; Wu et al., 2017). Low-grade and persistent inflammation contribute to progression of experimental and clinical hypertension; C-reactive protein levels are associated with deterioration of hypertension, suggesting that hypertension is in part an inflammatory disorder (Sesso et al., 2003; Vongpatanasin et al., 2007). Hypertension is often accompanied by autonomic dysfunction, which is detrimental to regulation of hemodynamics; on the other hand, cholinergic stimulation via inhibition of acetylcholinesterase appears to benefit autonomic control and inhibits inflammatory responses (Hilderman et al., 2004; Soares et al., 2004; Nolan et al., 2012).

Based on these findings, we hypothesized that CAP stimulation in AngII-induced hypertensive rats would promote beneficial adaptations in terms of autonomic control and hemodynamics. Therefore, the aim of this study was to determine whether alterations of CAP would alter inflammatory responses and fibrosis in the kidney as well as changes in BRS indices. A secondary aim was to elucidate the underlying mechanisms behind CAP in AngII-induced hypertension.

## Materials and Methods

### Ethics

All animal experiments were performed in accordance with the Animal Ethics Committee of Wenzhou medical university (Number: wydw2014-0058). Male Sprague-Dawley rats (specific pathogen-free class, weight 200–225°g, 6–8°weeks, Slac Laboratory Animal Center, Shanghai, China) were used in our experiments. Rats were treated according to the Guide for the Care and Use of Laboratory Animals by the National Institutes of Health (NIH).

### AngII Infusion and Treatments

**FIGURE 1:**
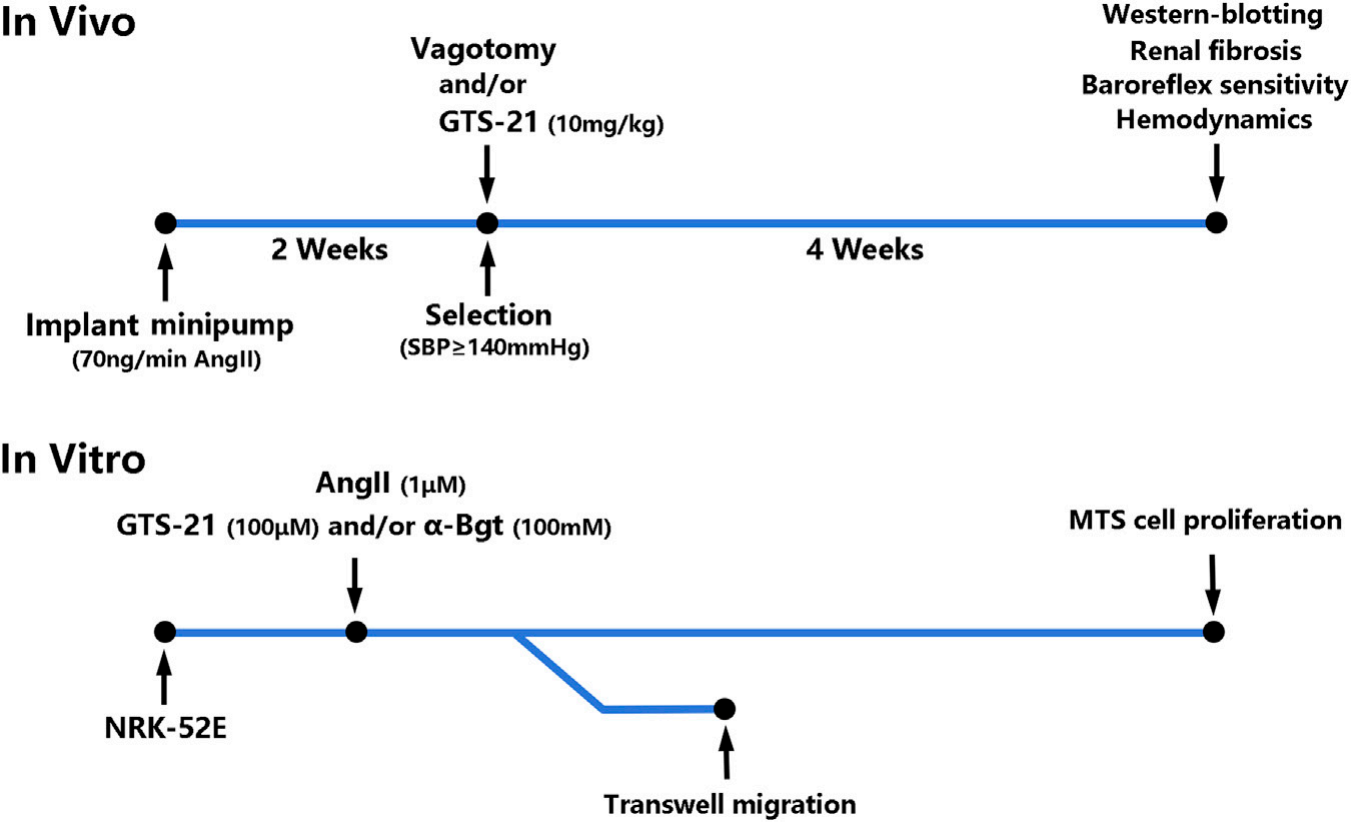
Timeline and flow chart of the complete set of experiments.

*In vivo* (Figure 1), Osmotic minipumps (Alzet Osmotic Pump, Model 2002; DURECT Corporation, United States) were placed in the dorsum of the neck under general anesthesia (2% Isoflurane/O_2_) for continuous infusion of AngII (350 ng/kg/min, Phoenix Pharmaceuticals, Burlingame, United States) for 2 weeks to induced hypertension according to previous study (Osmond et al., 2014). The rats that developed hypertension (systolic blood pressure ≥ 140 mmHg) were selected and divided into six groups randomly: 1) Sham-infused rats (0.9% NaCl) (Sham, *n* = 6); 2) Sham rats with vagotomy (Sham + Vag, *n* = 6); 3) AngII-induced hypertensive rats (Ang, *n* = 6); 4) hypertensive rats with vagotomy (Ang + Vag, *n* = 6); 5) hypertensive rats with GTS-21 administration (Ang + GTS, *n* = 6); 6) hypertensive rats with vagotomy and GTS-21 administration (Ang + GTS + Vag, *n* = 6). Unilateral cervical vagotomy (right-sided) was performed after hypertension confirmed (the blood pressure was measured by non-invasive blood pressure meter after AngII infusion). To perform unilateral cervical vagotomy, the vagi were exposed unilaterally in the neck, posterior to the carotid artery and the jugular vein, then right-sided vagus nerve was separated from the sympathetic trunk, and was secured under sterile conditions by a ceramic scissor (to avoid nerve stimulation) with a loop of 5-0 silk suture for ligation. Rats were allowed to recover for 2°days prior to subsequent processing. Rats received daily intraperitoneal injection of GTS-21 (10 mg/kg in saline, HY-14564A, MedChemExpress, China) (Yeboah et al., 2008) after vagotomy and continued for 4°weeks.

*In vitro* (Figure 1), the rat renal tubular epithelial cells line (NRK-52E) was obtained from the Cell Bank of the Chinese Academy of Sciences. Cells were cultured (37°C, 5% CO_2_) in low glucose Dulbecco’s modified Eagle’s medium (DMEM, Gibco BRL, United States) supplemented with 10% heat-inactivated fetal bovine serum (FBS), 100°U/ml penicillin, 100 μg/ml streptomycin sulfate, and 2 mmol/L L-glutamine. The NRK-52E cells were cultured into 6-well plates at a density of 2 × 10^5^ cells/well. They were divided into six groups: 1) Sham-treated (PBS) NRK-52E cells (Con); 2) Sham-treated NRK-52E cells with α-Bgt (Con + α-Bgt); 3) AngII-treated NRK-52E cells (Ang); 4) AngII-treated NRK-52E cells with α-Bgt (Ang + α-Bgt); 5) AngII-treated NRK-52E cells with GTS-21 administration (Ang + GTS); 6) AngII-treated NRK-52E cells with α-Bgt and GTS-21 administration (Ang + GTS+α-Bgt). For experiments using α-Bgt, the selective α7-AChR antagonist was added to the cell cultures 2 h before GTS-21 and AngII. And 30 min after the addition of the GTS-21, AngII was added to the cultures.

### Blood Pressure and ECG Monitoring

The rats were anesthetized (pentobarbital sodium, 30 mg/kg, intraperitoneally) after 4-weeks GTS-21 treatment. A pressure transducer was inserted into carotid artery (right-sided) for 1.0–1.5 cm to monitor blood pressure. A telemetry transmitter (HD-S11, DSI PhysioTel™, United States), which connected to the pressure transducer and two biopotential leads (two electrodes were embedded in the left upper limb and right lower limb respectively) was implanted in the peritoneum for recording blood pressure and ECG. Rats were allowed to recover for 1°day prior to record of digitized signals, and then housed for 3°days in cages with bottoms fitted with receivers (RPC-1 Single Receiver, DSI PhysioTel™, United States). The ECG signals and digitized blood pressure were analyzed by LabChart Pro Blood Pressure Analysis Module (AD Instruments, United States).

### BRS Measurement

Spontaneous BRS was calculated from 5 min segments of R-R interval (RRI) and mean blood pressure (MBP) data simultaneously. BRS was determined by analyzing data with Nevrokard SA-BRS software (Nevrokard, Slovenia) in the sequence method according to previous studies (Henze et al., 2008; Henze et al., 2013). Gain was determined as the average slope of linear regressions obtained from a minimum of three sequences that satisfied the following constraints: three or more consecutive RRIs with variation in the same direction, >0.5 ms that correlated (r^2^ > 0.85) with mean arterial pressure (MAP) variations of 0.5 mmHg, and with a three-beat delay. Coherence between RRI and MBP variability was determined as the square root of the ratio of the RRI and MBP power spectra with a segment length of 128 points, 50% overlap, and zero padding of 8. The average coherence in the LF and HF domains was calculated as the area under the curve within the specified frequency domains.

### Masson Stain

The kidney was isolated and embedded in paraffin. Samples were sectioned into 4 or 5 μm-thick slices for Mallory trichrome staining. The fibrosis index (%) was normalized by the total area of extracellular matrix. The views of sections under the microscope were chosen randomly and the index of fibrosis size (%) was calculated by an experienced technician who was blinded to the study groups. The fibrosis area was measured using ImageJ (National Institutes of Health, United States).

### Transwell Migration and MTS Cell Proliferation

In transwell migration experiment, a total of 1 × 10^6^ cells were added to the top chamber of 24-well transwell plates in 100 µl medium with 0.5% FBS, while the bottom filled with 600 ml medium with 10% FBS. After 6 h incubation, 4% paraformaldehyde was applied to fix the cells for 30 min, then staining these cells by crystal violet (0.1%) for 20 min. The upper surface of the filters was carefully wiped with a cotton-tipped applicator. The number of cells passing through the membrane was counted randomly in 5 non-overlapping 10x fields. For MTS cell proliferation, cells were plated in wells of 96-well plate and cultured for 24 h to reach steady state. Cells were incubated with AngII (1 μM) (Zhang et al., 2020), GTS-21 (100 μM) (Yue et al., 2015) and/or α-Bgt (Yue et al., 2015; Wu et al., 2017) for 12, 24, 36, and 48 h. The CellTiter 96® AQueous One Solution Reagent (20 µL, Promega, United States) was directly added to the culture wells after incubation, and incubated for another 2.5 h. The absorbance was recorded at 490 nm with a VersaMax microplate reader (Molecular Device GmbH, Germany).

### Immunofluorescence

After 72 h treatment of AngII, GTS-21 and/or α-bungarotoxin, NRK-52E cells were fixed with 4% paraformaldehyde (Solarbio, China) and permeabilized with Triton X100 (Solarbio, China). The cell slides were then blocked with 5% BSA (Solarbio, China) and incubated with primary antibodies at 4°C overnight. After washing three times with PBS, the corresponding fluorescent secondary antibodies (FITC-conjugated goat anti-mouse IgG, Beyotime, China; FITC-conjugated goat anti-rabbit IgG, Beyotime, China; TRITC-conjugated goat anti-rabbit IgG, Bioworld, China) was added to incubate for 1 h. After nuclear counterstaining with DAPI (Beyotime, China), images were examined under a fluorescence microscope (DP72, Olympus, Japan).

### Western Blot Analysis

Kidney tissues and cells were lyzed in ice-cold lysis buffer system containing RIPA lysate (1 ml, 89,900, ThermoFisher, China), protease inhibitors (10 μl, ST506–2, Beyotime, China), and phosphatase inhibitors (10 μl, P1260, Applygen, China) and centrifuged at 4°C at 12,000 rpm for 25–30 min. Protein sample extraction, concentration normalization, and detection of specific protein were conducted according to our previous description (Wu et al., 2017). The details of primary antibodies for immunofluorescence and western blot analysis are listed below: IL-1β (ab9722, Abcam, China), TNF-α (ab6671, Abcam, China), Collagen-I (14695-1-AP, Proteintech, China), α-SMA (ab32575, Abcam, China), E-cadherin (ab231303, Abcam, China), TGF-β (ab215715, Abcam, China), CNN2 (21073-1-AP, Proteintech, China), PAI-1 (ab66705, Abcam, China), *p*-smad2 (ab188334, Abcam, China), smad (ab40855, Abcam, China), MCP-1 (ab25124, Abcam, China), NGAL (ab63929, Abcam, China), *p*-IκB (#2859, CST, United States), IκB (#4812, CST, United States), Cytokeratin 8 (ab59400, Abcam, China), p-65 (ab16502, Abcam, China), p-P65 (Ser536; ab76302, Abcam, China), β-actin (60008-1-Ig, Proteintech, China).

### Statistical Methods

The data obtained from the experiment were expressed as mean ± standard deviation (SD). Shapiro-Wilk test and Kolmogorov-Smirnov test were used for normal distribution. The mean value of each group was compared with one-way analysis of variance (ANOVA) followed by Dunnett’s multiple-comparison test. Continuous data of cell proliferation were compared across time by two-way repeated measures ANOVA followed by Bonferroni correction post testing. A value of *p* < 0.05 was considered significant.

## Results

### Activated CAP Attenuated AngII-Induced Hypertension

**FIGURE 2:**
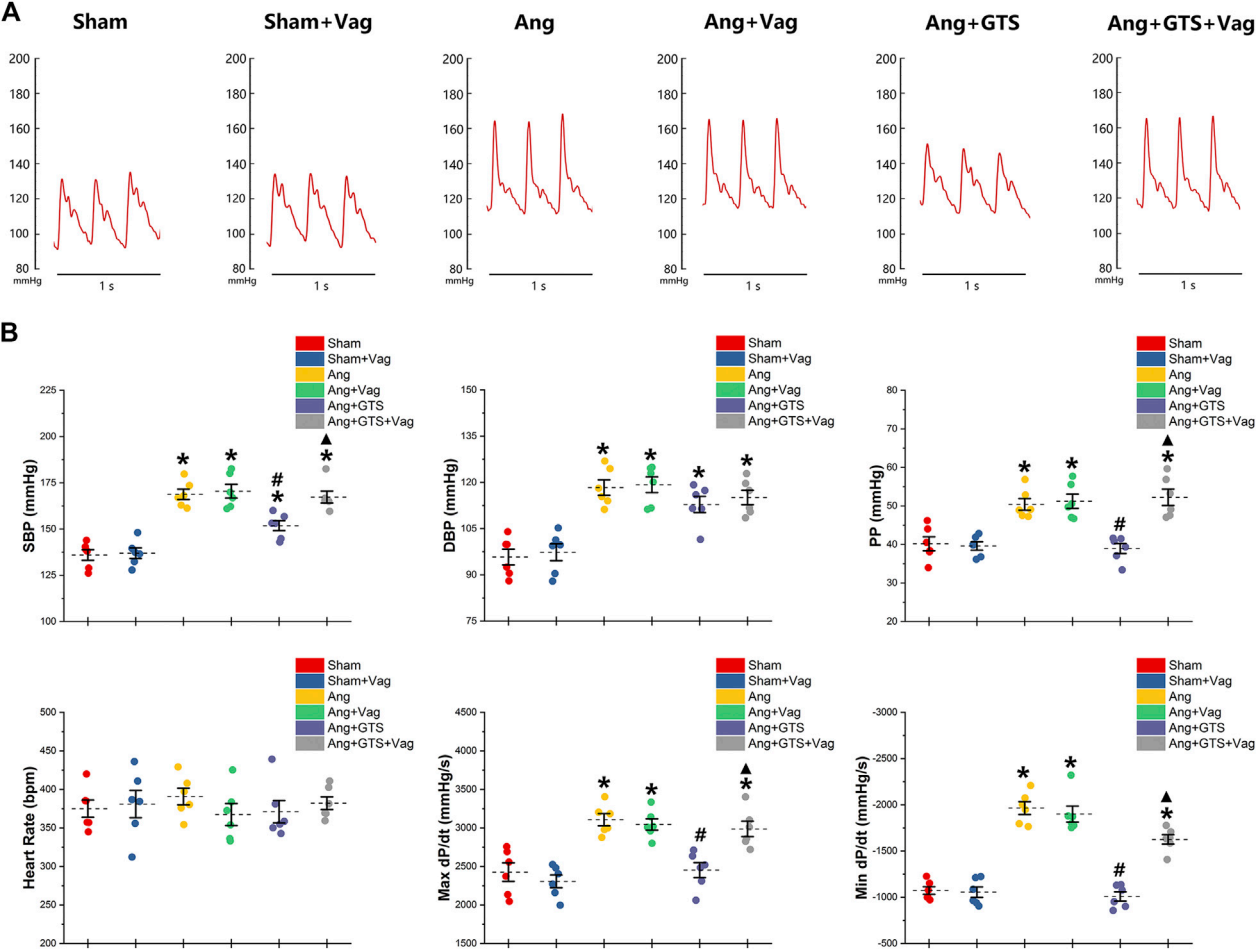
Treatment with GTS-21 attenuated AngII-induced hypertension. **(A)**, representative pressure waves of arterial pressure. **(B)**, statistic results of peripheral blood pressure parameters. Systolic blood pressure (SBP), diastolic blood pressure (DBP), pulse pressure (PP), heart rate, max dP/dt and min dP/dt were similar between sham rats irrespective of vagotomy. Higher SBP, DBP, PP, max dP/dt and min dP/dt were found in the Ang group than in the Sham group. GTS-21 reduced SBP, PP, max dP/dt and min dP/dt in AngII-infused rats, while Ang and Ang + GTS group shared similar DBP. Vagotomy performed previously partly attenuated the effects of GTS-21 on peripheral blood pressure. *p < 0.05 vs. Sham group, ^#^ *p* < 0.05 vs. Ang group, ^▲^ *p* < 0.05 vs. Ang + GTS group (*n* = 6 for each group).

Blood pressure were recorded by a pressure transducer and continuously monitored for 3 days. There were no significant differences in heart rate among the six groups (*p* > 0.05). Systolic blood pressure (SBP), diastolic blood pressure (DBP), pulse pressure (PP), heart rate, max dP/dt (maximal left ventricular pressure rising rate) and min dP/dt (maximal left ventricular pressure decline rate) were similar between sham rats irrespective of vagotomy (Sham vs. Sham + Vag, *p* > 0.05) (Figure 2; Table 1). 14-days AngII infusion significantly increased SBP, DBP, PP, max dP/dt and min dP/dt (Sham vs. Ang; SBP, 135.98 ± 6.33 vs. 168.76 ± 6.30 *p* < 0.05; DBP, 95.80 ± 5.77 vs. 118.35 ± 5.61 *p* < 0.05; PP, 40.19 ± 4.02 vs. 50.41 ± 3.41 *p* < 0.05; max dP/dt, 2,425.89 ± 267.31 vs. 3,106.50 ± 175.76 *p* < 0.05; min dP/dt, −1,071.84 ± 91.25 vs. −1964.34 ± 153.81 *p* < 0.05). GTS-21 reduced SBP, PP, max dP/dt and min dP/dt in AngII-infused rats, while Ang and Ang + GTS group shared similar DBP (Ang + GTS vs. Ang; SBP, 151.80 ± 6.08 vs. 168.76 ± 6.30 *p* < 0.05; PP, 38.95 ± 2.93 vs. 50.41 ± 3.41 *p* < 0.05; max dP/dt, 2,452.81 ± 215.88 vs. 3,106.50 ± 175.76 *p* < 0.05; min dP/dt, −1,008.81 ± 110.67 vs. −1964.34 ± 153.81 *p* < 0.05). Vagotomy performed previously partly attenuated the effects of GTS-21 on peripheral blood pressure (Ang + GTS vs. Ang + GTS + Vag; SBP, 151.80 ± 6.08 vs. 167.33 ± 7.15 *p* < 0.05; PP, 38.95 ± 2.93 vs. 52.23 ± 4.76 *p* < 0.05; max dP/dt, 2,452.81 ± 215.88 vs. 2,986.45 ± 223.69 *p* < 0.05; min dP/dt, −1,008.81 ± 110.67 vs. −1,625.05 ± 114.79 *p* < 0.05).

**TABLE 1:** Hemodynamics parameters of rats.

	Sham	Sham + Vag	Ang	Ang + Vag	Ang + GTS	Ang + GTS + Vag
SBP (mmHg)	135.98 ± 6.33	136.94 ± 6.27	168.76 ± 6.30*	170.45 ± 8.28*	151.80 ± 6.08*^#^	167.33 ± 7.15*▲
DBP (mmHg)	95.80 ± 5.77	97.35 ± 6.12	118.35 ± 5.61*	119.24 ± 5.71*	112.85 ± 5.81*	115.10 ± 5.20*
PP (beat/min)	40.19 ± 4.02	39.59 ± 2.44	50.41 ± 3.41*	51.21 ± 4.11*	38.95 ± 2.93^#^	52.23 ± 4.76*▲
Heart Rate (beat/min)	374.89 ± 25.07	380.89 ± 39.44	390.70 ± 24.04	367.38 ± 31.56	370.99 ± 32.63	382.00 ± 18.69
dP/dt_Max_ (mmHg/s)	2,425.89 ± 267.31	2,305.84 ± 185.47	3,106.50 ± 175.76*	3,043.61 ± 164.80*	2,452.81 ± 215.88^#^	2,986.45 ± 223.69*▲
dP/dt_Min_ (mmHg/s)	−1,071.84 ± 91.25	−1,055.11 ± 127.72	−1,964.34 ± 153.81*	−1,899.58 ± 193.53*	−1,008.81 ± 110.67^#^	1,625.05 ± 114.79*▲

Ang group got higher SBP, DBP, PP, max dP/dt and min dP/dt than Sham group. GTS-21 reduced SBP, PP, max dP/dt and min dP/dt in AngII-infused rats. Vagotomy attenuated the effects of GTS-21 on hemodynamics changes. *p < 0.05 vs. Sham group, ^#^ *p* < 0.05 vs. Ang group, ^▲^ *p* < 0.05 vs. Ang + GTS group. (Data are expressed as means ± SD, *n* = 6 for each group).

### Activated CAP Improved Autonomic Control

**FIGURE 3:**
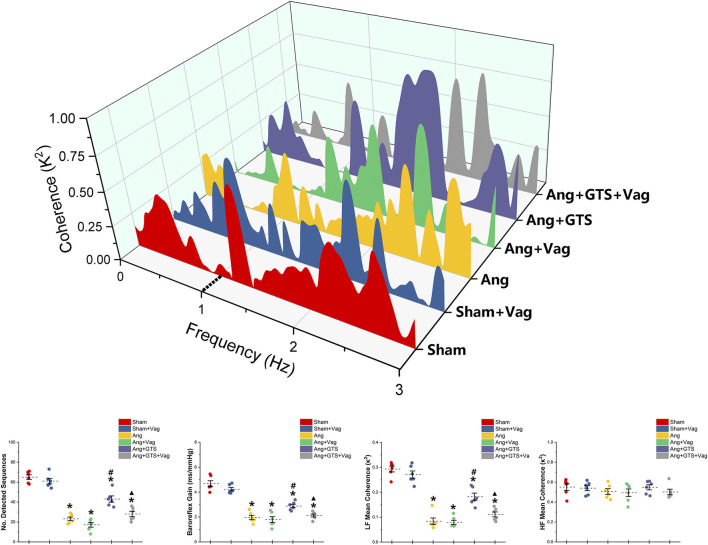
Treatment with GTS-21 improve baroreflex sensitivity in AngII-induced hypertension. The number of sequences (No. Detected Sequences) and the average gain of detected sequences (Baroreflex Gain) in AngII-infused rats were significantly lower than placebo-treated sham rats. AngII reduced LF mean coherence in the Ang group with no changes of HF mean coherence. Activating CAP by GTS-21 restore baroreflex sensitivity, showing as increased the number of sequences and average gain in the Ang + GTS group. Vagotomy significantly weakened the effects of GTS-21 on baroreflex sensitivity. *p < 0.05 vs. Sham group, ^#^ *p* < 0.05 vs. Ang group, ^▲^ *p* < 0.05 vs. Ang + GTS group (*n* = 6 for each group).

Charts of coherence between RRI and MAP variability of 0–3 Hz for representative rats and baroreflex analysis are shown in Figure 3. As expected, the number of sequences (No. Detected Sequences) that display baroreflex control and the average gain of detected sequences (Baroreflex Gain) in AngII-infused rats were significantly lower than placebo-treated sham rats (Sham vs. AngII; No. Detected Sequences, 65.00 ± 5.10 vs. 23.17 ± 4.10 *p* < 0.05; Baroreflex Gain, 4.68 ± 0.53 vs. 1.96 ± 0.39 *p* < 0.05). In addition, AngII reduced LF mean coherence in the Ang group with no changes of HF mean coherence (Sham vs. AngII; LF; 0.29 ± 0.03 vs. 0.08 ± 0.03 *p* < 0.05). Activating CAP by GTS-21 restore baroreflex sensitivity (Ang + GTS vs. AngII, No. Detected Sequences, 43.00 ± 7.12 vs. 23.17 ± 4.10 *p* < 0.05; Baroreflex Gain, 2.88 ± 0.31 vs. 1.96 ± 0.39 *p* < 0.05; LF; 0.18 ± 0.03 vs. 0.08 ± 0.03 *p* < 0.05). Interestingly, vagotomy significantly weakened the effects of GTS-21 on baroreflex sensitivity (Ang + GTS vs. Ang + GTS + Vag, No. Detected Sequences, 43.00 ± 7.12 vs. 28.00 ± 5.97 *p* < 0.05; Baroreflex Gain, 2.88 ± 0.31 vs. 2.12 ± 0.26 *p* < 0.05; LF; 0.18 ± 0.03 vs. 0.11 ± 0.02 *p* < 0.05).

### Activated CAP Inhibited AngII-Induced Renal Fibrosis

**FIGURE 4:**
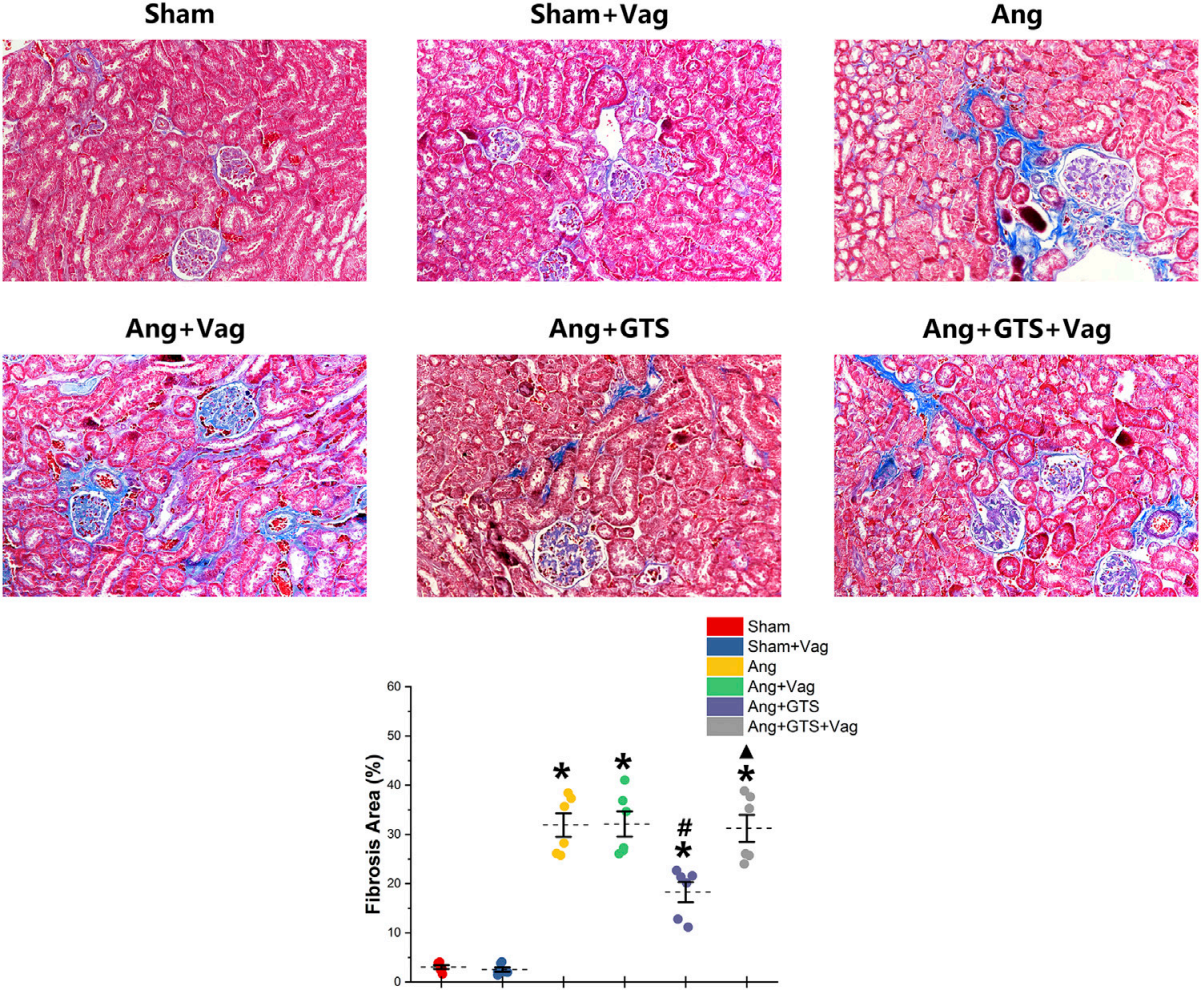
Treatment with GTS-21 inhibited renal fibrosis in AngII-induced hypertension. More fibrosis developed in renal tissue in the Ang group than in the Sham group. GTS-21 treatment, which activated CAP, shrank the fibrotic area. Vagotomy attenuated the effect of GTS-21 on fibrosis. *p < 0.05 vs. Sham group, ^#^ *p* < 0.05 vs. Ang group, ^▲^ *p* < 0.05 vs. Ang + GTS group (*n* = 6 for each group).

**FIGURE 5:**
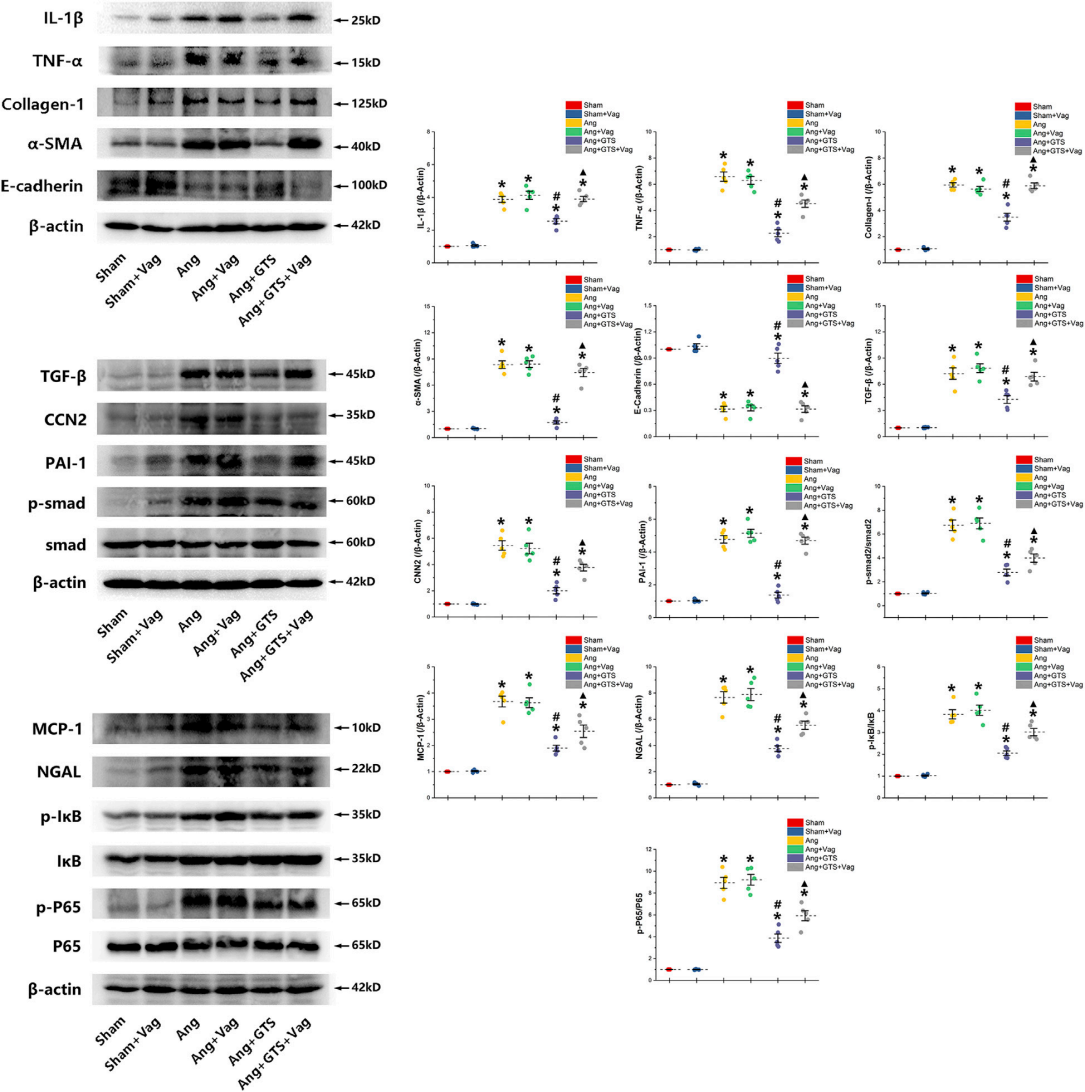
Western blotting demonstrated protein expression in EMT related biomarkers, pro-inflammatory factors, pro-fibrotic factors and activation of smad and NF-κB in renal tissue. GTS-21 increased the expression of E-cadherin in the Ang + GTS group. GTS-21 also reduced the EMT related biomarkers, pro-inflammatory factors and pro-fibrotic factors. There was less activation of the NF-κB and smad pathway in the Ang + GTS group than in the Ang group. Vagotomy partly inhibited the effect of GTS-21 on EMT related biomarkers, pro-inflammatory factors, pro-fibrotic factors and smad and NF-κB signal pathway. *p < 0.05 vs. Sham group, ^#^ *p* < 0.05 vs. Ang group, ^▲^ *p* < 0.05 vs. Ang + GTS group (*n* = 5 for each group).

**FIGURE 6:**
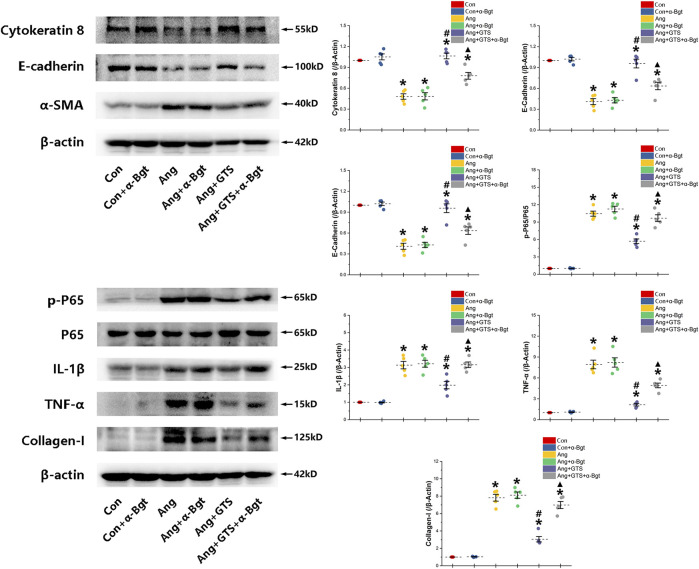
Western blotting demonstrated protein expression in EMT related biomarkers, pro-inflammatory factors, pro-fibrotic factors and NF-κB signal pathway in NRK-52E cells. AngII decreased expression of cytokeratin 8 and E-cadherin and increased α-SMA and collagen-I in NRK-52E cells. GTS-21 inhibited the expression of α-SMA in the Ang group and promote the expression of cytokeratin 8 and E-cadherin. And GTS-21 inhibited AngII-induced phosphorylation of NF-κB p65, pro-inflammatory cytokines and fibrotic medium (collagen-I) which are promoted by AngII. α-Bgt blunted the effect of GTS-21 on EMT related biomarkers, pro-inflammatory cytokines, fibrotic mediums and NF-κB phosphorylation. *p < 0.05 vs. Con group, ^#^ *p* < 0.05 vs. Ang group, ^▲^ *p* < 0.05 vs. Ang + GTS group (*n* = 5 for each group).

The AngII-induced renal fibrosis response were accessed using Masson’s trichrome staining and Western blot analysis *in vivo* and *vitro* (Figures 4–6). Significantly more fibrosis developed in the extracellular matrix in the Ang group than in the Sham group (Sham vs. AngII; Fibrosis area (%); 3.05 ± 0.87 vs. 31.92 ± 5.33 *p* < 0.05). GTS-21 treatment, which activated CAP, shrank the fibrotic area (Ang + GTS vs. AngII; Fibrosis area (%); 18.27 ± 4.54 vs. 31.92 ± 5.33 *p* < 0.05). Same changes also had been shown in the western blot result of pro-fibrotic factors (collagen-I, TGF-β, CNN2, PAI-1). Vagotomy attenuated the effect of GTS-21 on fibrosis (Ang + GTS vs. Ang + GTS + Vag; Fibrosis area (%); 18.27 ± 4.54 vs. 31.24 ± 6.11 *p* < 0.05).

### Activated CAP Inhibited Epithelial–Mesenchymal Transition in NRK-52e Cells

**FIGURE 7:**
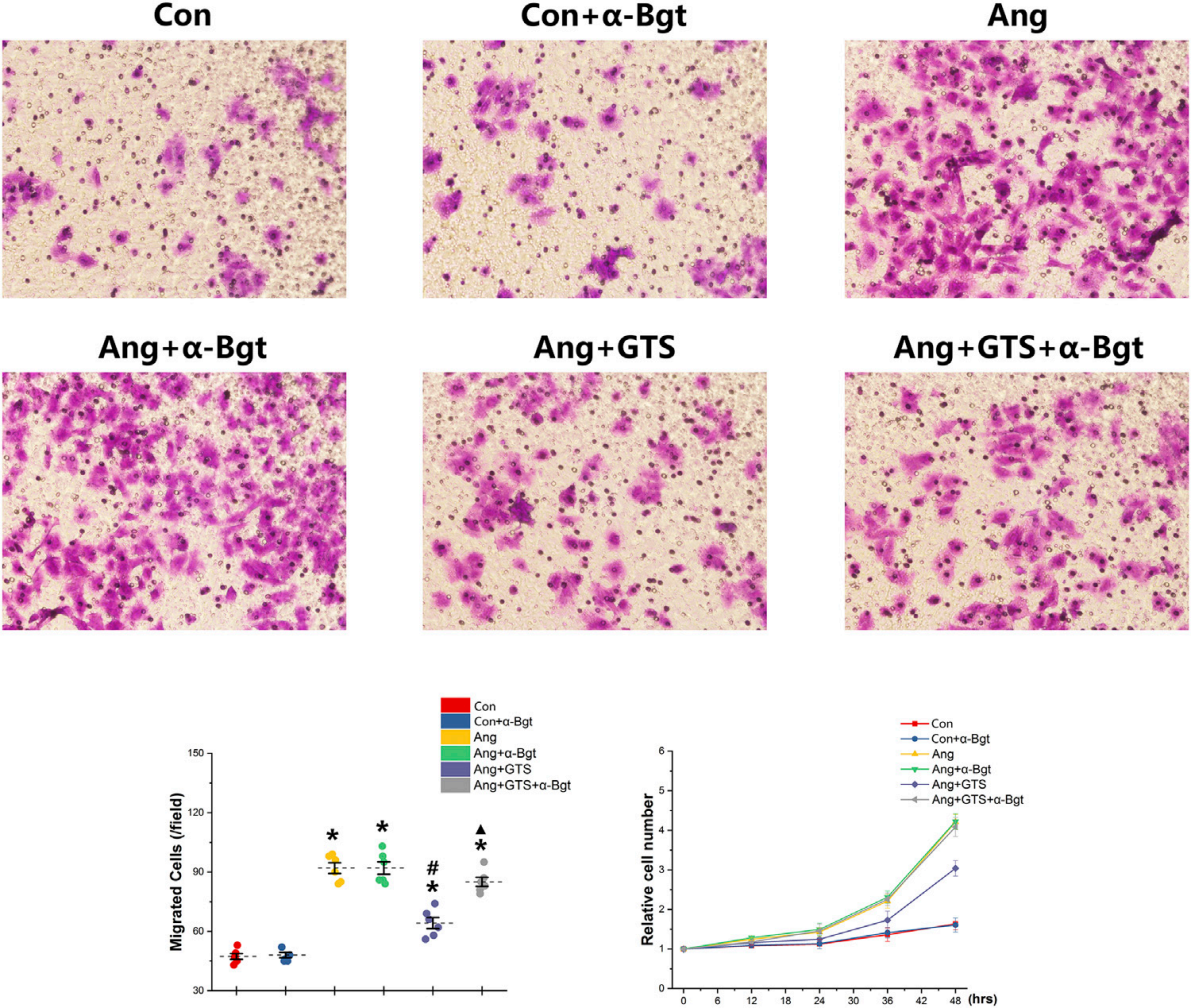
Treatment with GTS-21 inhibited transwell migration and MTS proliferation in NRK-52E cells. Significantly more transwell migration **(A, B)** and higher MTS cell proliferation **(C)** of NRK-52E cells were found in the Ang treatment group than in the Con group. Activating CAP by CTS-21 inhibited AngII-promoted transwell migration and MTS cell proliferation of NRK-52E cells. α-Bgt attenuated the effect of GTS-21 on transwell migration and MTS cell proliferation. *p < 0.05 vs. Con group, ^#^ *p* < 0.05 vs. Ang group, ^▲^ *p* < 0.05 vs. Ang + GTS group (*n* = 6 for repetition).

**FIGURE 8:**
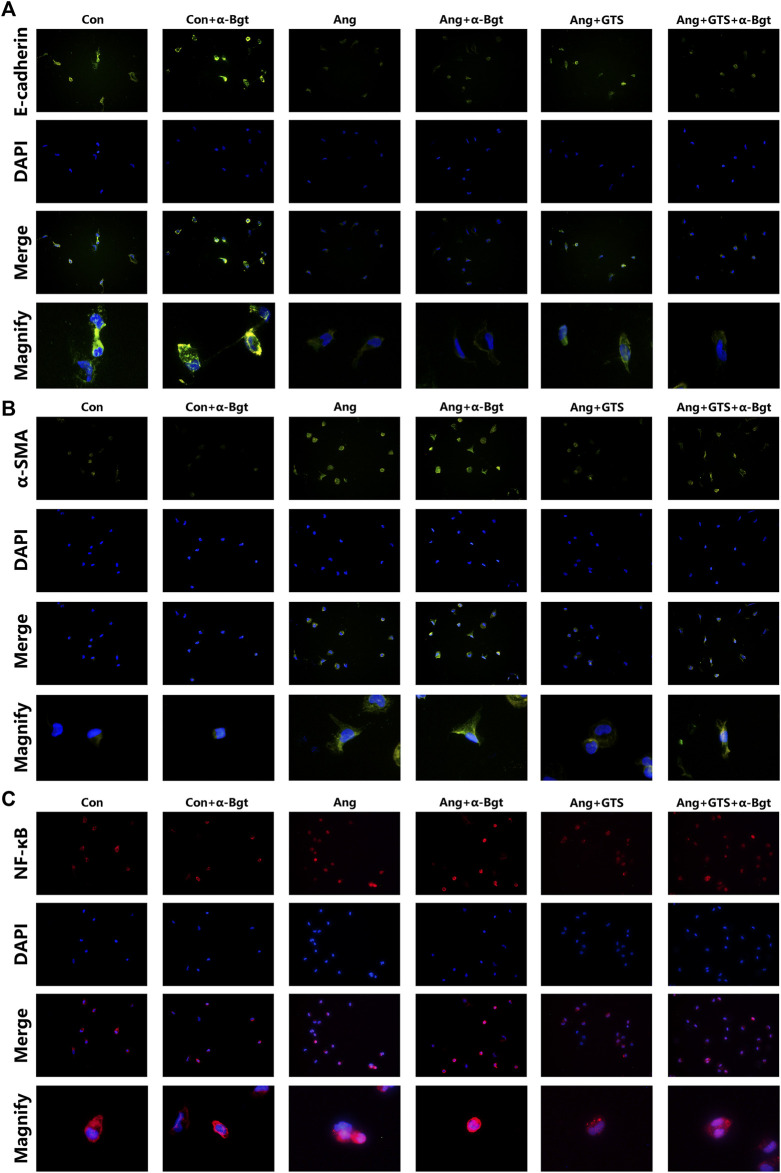
Treatment with GTS-21 inhibited NF-κB activation, decreased α-SMA expression and increased E-cadherin expression in AngII-stimulated NRK-52E cells. **(A)** GTS-21 significantly suppressed the nuclear translocation of AngII-induced NF-κB p65, and α-Bgt attenuated the effect of GTS-21 on suppressing NF-κB activation, **(B)** GTS-21 significantly increased the expression of E-cadherin in Ang + GTS group, and α-Bgt attenuated the effect of GTS-21 on E-cadherin expression, **(C)** increased expression of α-SMA was found in NRK-52E cells after AngII treatment. GTS-21 inhibited α-SMA expression, while α-Bgt weakened this effect (*n* = 3 for repetition).

We estimated the important biomarkers of Epithelial–mesenchymal transition (EMT) in the kidney, as EMT an important mechanism of renal interstitial fibrosis. In the results of present study, we found that CAP, which activated by CTS-21 treatment, inhibited transwell migration and MTS cell proliferation of NRK-52E cells (Figure 7) (Ang + GTS vs. Ang; Migrated cells (/field), 64.17 ± 6.22 vs. 92.00 ± 6.03 *p* < 0.05; Relative cell number, *p* < 0.05). Through the western blot analysis and immunofluorescent staining, we also found decreased expression of cytokeratin 8 and E-cadherin (both are renal tubular epithelial marker) and increased expression of α-SMA (fibroblast marker) and collagen-I after AngII treatment *in vivo* and *in vitro* (Figures 5, 6, 8A,B) (Sham vs. Ang/Con vs. Ang, *p* < 0.05). GTS-21 could significantly inhibit the expression of collagen-I and α-SMA in the Ang group and promote the expression of cytokeratin 8 and E-cadherin (Ang + GTS vs. Ang, *p* < 0.05). There results confirmed the potential role of CAP in inhibiting interstitial fibrosis in AngII-induced renal injury. Vagotomy or α-Bgt attenuated the effect of GTS-21 on EMT (Ang + GTS vs. Ang + GTS + α-Bgt, *p* < 0.05).

### Activated CAP Supressed Samd and NF-κB Activation

NF-κB is a transcriptional regulator that has marked effects on inflammatory response and fibrosis, and dysregulation of TGF-β/smad signaling is a possible pathogenic mechanism of renal fibrosis. As shown in Figure 8C (NRK-52E cells), Figure 5 (renal tissue) and Figure 6 (NRK-52E cells), the results of immunofluorescent staining and western bolt analysis showed that GTS-21 significantly inhibited AngII-induced nuclear translocation of NF-κB p65 and blocked the phosphorylation of Ser536 of NF-κB p65 that was stimulated in the Ang group *in vivo* and *vitro* (Ang + GTS vs. Ang, *p* < 0.05). GTS-21 also inhibited the smad pathway which are activated by AngII in renal tissue (Ang + GTS vs. Ang, *p* < 0.05). Vagotomy partly inhibited the effect of GTS-21 on smad and NF-κB signal pathway. α-Bgt significantly attenuated the effect of GTS-21 on inhibiting NF-κB activation and subsequent fibrotic and inflammatory responses in NRK-52E cells, suggesting an important role of α-7nAChR on CAP-mediated renal protection (Ang + GTS vs. Ang + GTS + α-Bgt, *p* < 0.05).

## Discussion

This is the first study to investigate the beneficial effects of CAP against the development of AngII-induced hypertension and renal injury. Our major finding is that, following 4-weeks CAP stimulation with GTS-21, changes in peripheral blood pressure were associated with changes in renal fibrosis and inflammatory responses and BRS in AngII-induced hypertension. The effects of CAP stimulation on AngII-induced hypertension depended on intact vagus nerves, suggested by the fact that vagotomy attenuated the effect of CAP on blood pressure and renal injury. This finding suggests an important role of the vagus nerve in the benefits mediated by CAP stimulation. EMT is an important mechanism of renal interstitial fibrosis, occurring in injured renal tubular epithelial cells and aggravating the progression of renal interstitial fibrosis (Zeisberg et al., 2008). AngII was used to induce NRK-52E cells to simulate EMT so as to evaluate the effect of CAP *in vitro*. We found that CAP stimulation slowed the process of EMT and inhibited renal fibrosis as well as inflammatory responses.

The renin-angiotensin-aldosterone system (RAAS) controls renal function and arterial pressure. As the main effector of the RAAS, AngII has both pro-inflammatory and vasoconstrictor effects on post-glomerular arteries, causing vascular and glomerular injuries and resulting in necrosis and fibrosis of the kidneys (Iversen and Ofstad, 1987; Mezzano et al., 2001; Murphy et al., 2015). AngII acts through its binding to two specific receptors, AT_1_ and AT_2_ (Ruiz-Ortega et al., 2003). The former is responsible for most of the pathophysiological actions and the latter is involved in cell growth inhibition and inflammatory cell recruitment in the kidney (Wolf et al., 1997; Ruiz-Ortega et al., 2001a; Ruiz-Ortega et al., 2003). AngII regulates profibrotic growth factors (e.g., TGF-β) mediating extracellular matrix accumulation via AT_1_. Blockade of TGF-β, induced by angiotensin-converting enzyme inhibitors or AT1 antagonists diminishes ECM production (Wolf et al., 1997). However, treatment strategies that target TGF-β have not achieved a promising effect with respect to TGF-β exerting anti-inflammatory properties (Grainger, 2004). AngII causes the adhesion of circulating cells to endothelial and mesangial cells as well as the migration of inflammatory cells into the kidney. The upregulation of adhesion molecules, cytokines, and chemokines was responsible for this process (Ruiz-Ortega et al., 2001b) AngII upregulates many proinflammatory genes through the activation of several intracellular signaling systems, nuclear factor-κB (NF-κB mainly) (Ruiz-Ortega et al., 2001b). AngII activates the renal NF-κB pathway that was partially diminished by AT_1_ or AT_2_ antagonists alone, and was abolished by combination of both receptor antagonists, suggesting that NF-κB activation was mediated by both AT_1_ and AT_2_ receptors (Ruiz-Ortega et al., 2001b; Esteban et al., 2004). NF-κB inhibition attenuated renal interstitial inflammation and hypertension in spontaneously hypertensive rats (Rodríguez-Iturbe et al., 2005). These data suggested that the combined blockade of both AT_1_ and AT_2_ receptors as well as inhibition of the NF-κB pathway is necessary to halt renal inflammation. Consistent with the results of a previous study, by blocking activation of NF-κB we found that inhibiting renal inflammatory responses and fibrosis limited renal injuries and improved peripheral hemodynamics.

The ideal anti-inflammatory responses, should be reversible, rapid, localized, free of side-effects, and adapted to changes in inflammatory input signals. CAP seems to be a perfect candidate platform to inhibit inflammatory responses because the nervous system is composed of sensory and motor systems that react rapidly to input inflammation. Rapid and localized efferent activity of the vagus nerve regulates cholinergic anti-inflammatory reflexes, the innate immune response. In CAP, inflammatory input signals monitor and adjust inflammatory responses by neuronal sensory pathways that deliver information to the nucleus tractus solitarius and dorsal motor nucleus; then activated efferent vagus nerve fibers suppress inflammatory responses through the release of acetylcholine (ACh) (Borovikova et al., 2000; Saeed et al., 2005). ACh has been reported to bind to the α7 nicotinic ACh receptor subunit (α7-nAChR) on cytokine-producing immune cells, and this binding inhibits the activation of NF-κB and the subsequent expression of a pro-inflammatory cascade (Parrish et al., 2008). Early researchers had access to the therapeutic potential of CAP in attenuating systemic inflammation caused by endotoxemia; several studies reported a beneficial effect of CAP in many inflammation-mediated diseases (Borovikova et al., 2000; Ghia et al., 2008; Tracey, 2009; Nolan et al., 2012). Current research widely believes that the JAK2/STAT3/NF-κB and PI3K/AKT/NF-κB signaling pathways are important pathways involved in the anti-inflammatory/anti-fibrotic effects of alpha-7 acetylcholinergic receptors (α7-nAChR) (Bertrand et al., 2015; Hoover, 2017). Dong et al. reported that the deficit of the CAP appears to contribute to the pathogenesis of end-organ damage in spontaneously hypertensive rats’ model, suggesting that CAP may be a target for preventing cardiovascular disease resulting from hypertension (Li et al., 2011). In a sham-controlled pilot trial, engaging the CAP by non-invasive stimulation of the vagus nerve for treatment of inflammatory musculoskeletal pain and fatigue in systemic lupus erythematosus (SLE) are promising (Aranow et al., 2020). GTS-21, one of the first reported ligands differing from nicotine to show binding specificity for the α7-nACh, and this compound is not solely selective for α7-nAChRs and also binds to α4β2-nAChRs, but with a lower affinity (Bertrand et al., 2015). GTS-21 mediated anti-inflammatory action depending on α-n7AChR; bioengineering knockdown of α-n7AChR blocked GTS-21-mediated inhibition of pro-inflammatory cytokines (Khan et al., 2012). GTS-21 has been characterized extensively both *in vitro* and *in vivo* and is one of the first molecules that was advanced into early phase clinical trials. Several studies have reported that GTS-21 was a quite effective immunomodulatory drug and could attenuated pancreatitis disease, improved the survival rate of sepsis, reduced the level of TNF-related endotoxin in the lung and attenuated LPS-induced renal injury (Bertrand et al., 2015; Gao et al., 2017). In this study, we demonstrate a strong and significant effect of CAP on AngII-induced renal inflammation and hypertension. Adding a cholinergic analogue, GTS-21 to the AngII-induced hypertension model inhibited the pro-inflammatory cytokine expression and fibrotic process in the kidney, suggesting a further potential for anti-inflammatory and anti-fibrotic effects in renal injury by exploring CAP modulation. Furthermore, in the *ex vivo* AngII-stimulated NRK-52E cell model, GTS-21 inhibited the expression of pro-inflammatory cytokines as well as the pro-fibrotic medium.

The dysfunction of autonomic activity, characterized by a reduced imbalance tone ratio of sympathetic/parasympathetic activity, were hypothesized to underlie the development of hypertension (Dauphinot et al., 2013). The arterial baroreflex acts against increases in blood pressure by inhibiting sympathetic activity, and baroreflex control is a mechanism responsible for short-term control of blood pressure. Hypertension has been consistently associated with the reduction in BRS and autonomic dysfunction in several clinical and basic research studies (Carthy, 2013; De Queiroz et al., 2013). The impairment of BRS predicts mortality in hypertension, and plays an indispensable role in the long-term development of hypertension (Huang et al., 2017). As predicted, we observed changes in BRS in the Ang group compared with Sham group. These changes were characterized by a reduced number of detected sequences, baroreflex gain and LF mean coherence in the Ang group, suggesting the loss of BRS and continued absence of vagal-mediated respiratory oscillations (Van De Borne et al., 1997; Henze et al., 2008). Previous studies suggested that averaging coherence across the LF domain is a more reliable method than HF domain for comparing BRS between groups (Pinna et al., 2002). Interestingly, in present study, no significant differences were found in HF mean coherence among six groups. Generally, the functional organization of neural-control immunoregulation (CAP) is based on principles of reflex regulation. The detection of pathogen fragments, cytokines, and other immune molecules by sensory neurons generates immunoregulatory responses through efferent autonomic neuron signaling. In detail, the neural mechanism relies upon activation of vagus nerve afferent sensory fibers that signal the brain that inflammation is occurring. Immunogenic stimuli activate vagal afferents either directly by cytokines from pro-inflammatory cells or indirectly through the chemoreceptive cells located in vagal paraganglia (Goehler et al., 2000). Nodose ganglion-located visceral vagus afferent fibers terminate primarily within the dorsal vagal complex (DVC) of the medulla oblongata. In the nucleus tractus solitaries (NTS), a part of DVC, the main portion of vagal sensory input is received by neurons coordinating autonomic function and interaction with the endocrine system, associated with the modulation of neurohormonal anti-inflammatory responses (Iversen et al., 2000). As a part of DVC, dorsal motor nucleus (DMN) is the major site of origin of preganglionic vagus efferent fibers, contributing to regulation of localized peripheral inflammation (Pavlov et al., 2003). Studies have reported that the neuro-immune effects of the CAP depends on the integrity of the entire reflex circuit. Partly blocking reflex circuit either at the afferent/efferent nerve or at the receptor/effector can inhibit CAP from exerting anti-inflammatory effects (Ghia et al., 2007; Rosas-Ballina et al., 2011). Therefore, in order to verify the importance of the integrity of the entire reflex circuit in this study, we performed unilateral vagotomy on rats in our study. In general, increasing vagal tone by vagus nerve stimulation exerts an anti-inflammatory effect through the CAP pathway (Borovikova et al., 2000); interestingly, in our study, directly activating CAP by selective α7-nAChR agonist GTS-21 improved the vagus nerve function (improvement of the BRS) in reverse, consistent with a previous study (Soares et al. reported that cholinergic stimulation with pyridostigmine increased heart rate variability and BRS) (Soares et al., 2004), confirming a close relationship between CAP and autonomic control. Moreover vagotomy attenuated the effect of CAP which activated by GTS-21 in our study. The current researches cannot explain this contradiction. Perhaps under certain circumstances, CAP reflex circuit has positive feedback characteristics, and perhaps the anti-inflammatory effect exerted by activating cholinergic receptors indirectly improves the function of the vagus nerve. Further studies are needed.

The activated NF-κB signaling pathway is an important contributor to the inflammatory response and fibrosis in renal injury (Liu, 2011; Zhou et al., 2017). The over-expression of inflammatory mediators and activation of the NF-κB signaling pathway stimulate pro-inflammatory cascade. Under normal physiological conditions, NF-κB is located in the cytoplasm bounding with IκB; once the pathway is activated, NF-κB translocated to the nucleus to mediate production of inflammatory cytokines (Baldwin, 1996). Studies have reported that the NF-κB signaling pathway activation promotes interstitial inflammation, that in turn leads to renal interstitial fibrosis; inhibition of NF-κB pathway activation delays renal fibrotic progression (Zhou et al., 2017). EMT in injured renal tubular epithelial cells plays an important role in the process of interstitial fibrosis (Zeisberg et al., 2008). During EMT, renal tubular epithelial cells lose the phenotype of epithelial cells, obtaining mesenchymal cell phenotype in the meanwhile, causing an increase of myofibroblasts (Zeisberg and Kalluri, 2004). Activated NF-κB translocates from the cytoplasm into the nucleus, triggering and advancing EMT progress (He et al., 2019). We found that AngII induced EMT in renal tubular epithelial cells and NRK-52E cells. It induced loss of intercellular epithelial adhesion molecule E-cadherin, which is an important determinant for maintenance of the epithelial phenotype (Tepass et al., 2000), and increased in the expression of α-SMA (a marker of myofibroblasts). The consequence was deposition of extracellular matrix and interstitial fibrosis, characterized by a significant increase in the expression of collagen-I protein.

We also used GTS-21 to determine the effect of CAP on AngII-induced renal injury. We found that activated CAP inhibited EMT and improved renal fibrosis both *in vivo* and *in vitro*. The α7-nAChR selective antagonist α-Bgt attenuated the anti-inflammatory and anti-fibrotic effects of GTS-21 on AngII-induced injury in NRK-52E cells by blocking the inhibition of NF-κB pathway activation, suggesting an important role of α7-nAChR in NF-κB pathway-mediated inflammatory response and fibrosis.

## Limitations

Although it is reasonable to infer that the renal protective effect of GTS-21 works involving the CAP pathway, unfortunately, our study only evaluates the changes in NF-κB and its downstream, and has not conducted in-depth research on the pathway mechanism involved between α7-nAChR receptor and NF-κB.

## Data Availability

The original contributions presented in the study are included in the article/Supplementary Material, further inquiries can be directed to the corresponding author.
