# Supplementary material for: Activation of the Cholinergic Anti-inflammatory Pathway Attenuated Angiotension II-Dependent Hypertension and Renal Injury
Source: Front Pharmacol. 2021 Mar 17;12:593682. doi: 10.3389/fphar.2021.593682 (PMC8010129; doi:10.3389/fphar.2021.593682)
Supplement: Supplementary file 1 [file table1.docx]

|  |  | Sham | Sham+Vag | Ang | Ang+Vag | Ang+GTS | Ang+GTS+Vag |
| --- | --- | --- | --- | --- | --- | --- | --- |
| After 2-week AngII infusion | SBP (mmHg) | 133.73±4.50 | 135.08±4.78 | 169.34±6.68 | 165.34±6.00 | 164.36±7.11 | 166.47±6.18 |
|  | DBP (mmHg) | 93.76±7.08 | 94.07±4.90 | 119.84±6.26 | 116.17±9.57 | 115.79±7.56 | 118.91±10.70 |
|  | PP  (beat/min) | 39.97±3.86 | 41.01±2.54 | 49.54±2.78 | 49.17±4.26 | 48.57±2.94 | 47.55±5.05 |
| After 4-week treatment | SBP (mmHg) | 135.98±6.33 | 136.94±6.27 | 168.76±6.30 | 170.45±8.28 | 151.80±6.08* | 167.33±7.15 |
|  | DBP (mmHg) | 95.80±5.77 | 97.35±6.12 | 118.35±5.61 | 119.24±5.71 | 112.85±5.81 | 115.10±5.20 |
|  | PP  (beat/min) | 40.19±4.02 | 39.59±2.44 | 50.41±3.41 | 51.21±4.11 | 38.95±2.93* | 52.23±4.76 |

**S-Table.1 Hemodynamic parameters of rats after 2-week AngII**

**infusion/after 4-week treatment**

***p<0.05 vs After 2-week AngII infusion**
